# Multi-omics integration and machine learning-driven construction of an immunogenic cell death prognostic model for colon cancer and functional validation of FCGR2A

**DOI:** 10.3389/fphar.2025.1746907

**Published:** 2026-01-22

**Authors:** Haipeng Wang, Ningning Chen, Weijia Wang

**Affiliations:** 1 Department of Medical Oncology, Shaanxi Provincial People’s Hospital, Xi’an, Shaanxi, China; 2 Department of Medical Oncology, The First Affiliated Hospital of Xi’an Jiaotong University, Xi’an, Shaanxi, China

**Keywords:** colon cancer, immunogenic cell death, machine learning, prognosis prediction, tumor immune microenvironment

## Abstract

**Background:**

Immunogenic cell death (ICD) influences tumor immune microenvironment remodeling and immunotherapy response. However, the prognostic value of ICD-related genes in colon cancer has not been systematically clarified. This study aimed to develop an ICD-based prognostic model and explore its association with the immune microenvironment and treatment sensitivity.

**Methods:**

Transcriptomic and clinical data of colon adenocarcinoma (COAD) patients were obtained from TCGA and GTEx, with GSE17538 and GSE38832 used as external validation cohorts. Single-cell RNA-seq data from the Colon Cancer Atlas were analyzed to characterize ICD-associated T-cell states. Differentially expressed genes between high and low ICD-score T cells were identified using ssGSEA, followed by WGCNA to select immune-related modules. One hundred seventeen machine-learning model combinations were evaluated to construct the optimal prognostic signature. Immune infiltration was assessed using CIBERSORT, ssGSEA, and ESTIMATE. GSEA explored pathway differences, while drug sensitivity was predicted using pRRophetic. The top-weighted gene was validated through *in vitro* assays.

**Results:**

Seven major cell types were identified within the tumor microenvironment. T cells with high and low ICD scores exhibited distinct functional and spatial patterns. WGCNA identified a key module highly correlated with ICD scores, and 51 genes were screened. The Random Survival Forest model yielded a 15-gene ICD-related signature with strong prognostic performance (C-index 0.968 in TCGA; 0.767 and 0.855 in validation cohorts). High-risk patients consistently showed poorer survival (*p* < 0.001). A combined nomogram demonstrated stable predictive accuracy. High-risk patients displayed increased M2 macrophages and Tregs, whereas low-risk patients exhibited higher activated CD4^+^ T cells and plasma cells. EMT and angiogenesis pathways were enriched in the high-risk group, while metabolic pathways predominated in the low-risk group. High-risk patients were more sensitive to drugs such as Dasatinib. FCGR2A overexpression promoted proliferation, migration, and invasion *in vitro*.

**Conclusion:**

The 15-gene ICD-based model effectively predicts COAD prognosis, reflects immune microenvironment heterogeneity, and offers insights for individualized treatment planning.

## 1 Introduction

Colon cancer is one of the most common malignant tumors worldwide, with its incidence ranking third among all cancer types and mortality ranking second (Bray et al., 2024). Despite continuous advances in comprehensive treatment modalities including surgical resection, chemotherapy, radiotherapy, and targeted therapy, the 5-year overall survival (OS) rate for colon cancer patients remains only 64%, with particularly grim prognosis for patients with advanced and metastatic colon cancer (Crooke et al., 2018). Although the traditional tumor-node-metastasis (TNM) staging system plays an important role in clinical decision-making, due to the high molecular heterogeneity of colon cancer, patients at the same stage often exhibit vastly different treatment responses and prognostic outcomes (Guinney et al., 2015; Gaiani et al., 2021). Therefore, there is an urgent need to develop more precise prognostic assessment tools and molecular classification systems to achieve individualized treatment and precision medicine for colon cancer patients (Dang et al., 2024).

In recent years, immunogenic cell death (ICD), as a critical bridge connecting tumor cell death and anti-tumor immune response, has attracted widespread attention in the field of cancer therapy (Kroemer et al., 2013). ICD is a unique mode of cell death characterized by the ability of dying or dead tumor cells to release damage-associated molecular patterns (DAMPs), including plasma membrane translocation of calreticulin, release of high mobility group box 1 (HMGB1), and secretion of adenosine triphosphate (ATP) (Galluzzi et al., 2020). These DAMP signals can be recognized and captured by antigen-presenting cells, subsequently promoting dendritic cell maturation, initiating and expanding tumor-specific T cells, and ultimately inducing potent adaptive immune responses (Krysko et al., 2012). ICD is not only an important mechanism through which certain chemotherapeutic drugs (such as anthracyclines and oxaliplatin) and radiotherapy exert anti-tumor effects but is also considered a key factor in improving the efficacy of immune checkpoint inhibitors (Garg et al., 2017; Zhu et al., 2020). Current research indicates that the expression levels of ICD-related genes are closely associated with tumor immunogenicity, immune cell infiltration patterns, and patient responsiveness to immunotherapy (Lisandrelli et al., 2025). Recent years have witnessed interest in ICD-related prognostic models across cancer types. In bladder cancer, Guo L. et al. (2024) established a seven-gene risk signature with high-risk subgroups showing worse prognosis. In hepatocellular carcinoma, Zhang et al. (2025) found high ICD scores corresponded to immune-excluded TME. In endometrial and cervical cancers, Yi et al. (2025) and Ning et al. (2025) developed prognostic models with five and seven ICD genes respectively, with low-risk patients showing stronger immunotherapy response. However, these studies have limitations including limited sample sizes, single algorithms, and lack of functional validation. Furthermore, systematic studies on ICD-related genes in colon cancer remain limited, and their expression patterns, biological functions, and value in prognosis prediction and treatment decision-making have not been fully elucidated, necessitating in-depth research (Catanzaro et al., 2025; Rodrigues et al., 2022).

Multi-omics integration analysis provides a powerful technical platform for comprehensively analyzing the role of ICD-related genes in colon cancer (Guo et al., 2020). Single-cell transcriptomics technology can accurately characterize the heterogeneity of various immune cells in the tumor microenvironment at single-cell resolution, identifying cell subpopulations with different ICD characteristics and their gene expression profiles (Valdes-Mora et al., 2018; Wu et al., 2021). Large-scale tumor transcriptomics data provide sufficient sample sizes for validating differential expression patterns and clinical relevance of ICD-related genes (Yu et al., 2024). Clinical omics data contain key information including patient survival information, pathological features, and treatment responses, serving as an important foundation for constructing prognostic prediction models and evaluating clinical value (Tran et al., 2025; Zhang et al., 2024). WGCNA can identify gene modules closely related to ICD immune phenotypes from a systems biology perspective, while machine learning algorithms, by integrating multiple modeling strategies, screen the most predictive feature gene combinations from numerous candidate genes to construct robust prognostic risk scoring models (Langfelder and Horvath, 2008).

This study innovatively established a multi-omics integrative analytical framework, that used single-cell transcriptomics to reveal, for the first time, the heterogeneity of ICD scores and specific gene expression profiles of T-cell subpopulations in colon cancer at single-cell resolution. By combining large-scale transcriptomic data and clinical omics information, the study systematically identified 15 core ICD-related immune genes and constructed a high-performance prognostic prediction model, elucidating the intrinsic relationship between ICD gene characteristics and the remodeling of the tumor immune microenvironment. This provides a molecular basis for explaining heterogeneous responses to immunotherapy in colon cancer (Kourou et al., 2015). At the clinical application level, the risk scoring model constructed can precisely identify high-risk patients with immunosuppressive microenvironment characteristics and low-risk patients with immune-activated profiles. This stratification can guide patient selection for immune checkpoint inhibitors and formulate individualized chemotherapy regimens based on drug sensitivity differences in different risk subgroups. Furthermore, functional validation of key genes such as FCGR2A lays the foundation for developing novel therapeutic targets, while the Nomogram model integrating clinical pathological parameters provides clinicians with a convenient and practical prognostic assessment and treatment decision-making tool with direct clinical translational value (Iasonos et al., 2008; Zhang et al., 2007).

## 2 Materials and methods

### 2.1 Data acquisition and preprocessing

RNA sequencing data (transcripts per million, TPM normalized) and corresponding clinical information for colon adenocarcinoma (COAD) were downloaded from the Cancer Genome Atlas (TCGA) database. The dataset included 420 tumor samples and 41 adjacent normal tissue samples. To increase the normal control sample size, RNA sequencing data from 779 normal colon tissue samples were obtained from the Genotype-Tissue Expression (GTEx) database and merged with TCGA normal samples as the control group (total 820 normal samples). To address potential batch effects arising from different data sources and platforms, we performed batch effect correction using the ComBat algorithm implemented in the R package sva (version 3.54.0). ComBat employs an empirical Bayes framework to estimate and remove systematic technical variations between batches while preserving biologically relevant variation. The batch-corrected expression matrix was used for all subsequent differential expression and downstream analyses. Only protein-coding genes were retained for subsequent analysis. For genes with multiple transcript variants, aggregation was performed by calculating average expression levels. Gene expression data underwent log2 transformation (log2(TPM+1)) to ensure normal distribution. Tumor samples were matched with clinical metadata, including age, TNM stage, pathological grade, sex, OS status, and OS time. Samples with OS time ≤ 0 days were excluded from survival analysis. Clinical staging information was standardized by removing subgroup identifiers and converting missing or ambiguous values to NA.

Additionally, two independent colon cancer cohort datasets were downloaded from the Gene Expression Omnibus (GEO) database for external validation. The GSE17538 dataset contained 232 patient samples with OS information, and the GSE38832 dataset contained 122 patient samples with disease-specific survival (DSS) information. The microarray expression data from these datasets underwent background correction, normalization, and log2 transformation, followed by integration with corresponding clinical follow-up data.

### 2.2 Single-cell RNA sequencing analysis

Single-cell RNA sequencing data for colon cancer were obtained from the Colon Cancer Atlas database, and the Read10X function in Seurat (v5.0.1) was used to load raw count matrices. Initial quality control retained genes expressed in ≥3 cells and cells expressing ≥200 genes. Quality control metrics were calculated for each cell, including detected gene count (nFeature_RNA), total unique molecular identifier (UMI) count (nCount_RNA), mitochondrial gene percentage (percent.mt), and ribosomal gene percentage (percent.ribo). Thresholds were set based on the distribution of quality control metrics for cell filtering: high-quality cells with nFeature_RNA between 200–6,000, nCount_RNA between 500 and 40,000, percent.mt <20%, and percent.ribo < 50% were retained. Filtered data were normalized and variance-stabilized using the SCTransform method.

T cells were specifically identified using canonical CD3 complex markers (CD3D, CD3E, CD3G). Cells were defined as T cells if they showed detectable expression (log-normalized expression >0) of at least two CD3 genes and lacked NK cell markers (NCAM1, KLRD1, NKG7) or ILC-specific markers. Additional T cell markers (CD4, CD8A, CD8B, LCK, IL7R) were used for validation. This approach ensures specific identification of *bona fide* T cells while excluding NK cells and ILCs from the original TNKILC population. Cell type annotation information was integrated from provided metadata files, including pre-computed t-distributed stochastic neighbor embedding (t-SNE) coordinates and three levels of cell clustering information. Annotation information was added to the Seurat object, and dimensionality reduction objects were created based on original t-SNE coordinates for visualization. T cell population cell barcodes were extracted based on major cell type annotations, a new Seurat object was created, and reclustering analysis was performed. Extracted cells underwent normalization (NormalizeData), highly variable gene identification (FindVariableFeatures), data scaling (ScaleData), and principal component analysis (RunPCA). The optimal number of principal components was determined through ElbowPlot, and selected principal components were used for cell clustering (FindNeighbors and FindClusters), t-SNE dimensionality reduction (RunTSNE), and uniform manifold approximation and projection (UMAP) dimensionality reduction (RunUMAP). Non-T cell subpopulations were excluded based on marker gene expression patterns, retaining pure T cell subpopulations for subsequent analysis.

The single-sample gene set enrichment analysis (ssGSEA) method was used to calculate ICD-related scores for each T cell. ICD-related gene sets were compiled from ImmPort and InnateDB databases (Supplementary Material), and ssGSEA analysis was implemented using the Gene Set Variation Analysis (GSVA) package, applying a Poisson kernel density function to raw count matrices. T cells were divided into high and low ICD score groups based on the median ICD score (Yi et al., 2025). Using ICD score grouping as the grouping variable, the FindAllMarkers function was used to identify differentially expressed genes between high and low ICD score T cell subpopulations.

### 2.3 Weighted gene Co-expression network analysis

The ssGSEA method was used to calculate immune scores for each sample in the TCGA cohort. Immune-related gene sets were integrated from the IMMPORT database and InnateDB database (Breuer et al., 2013). Genes expressed in the TCGA dataset were extracted from immune-related differential genes identified in single-cell analysis, and genes with zero standard deviation were filtered out. The WGCNA package was used for co-expression network analysis. First, data quality was checked through the goodSamplesGenes function, and outlier samples were detected using hierarchical clustering and sample distance matrices.

The soft-thresholding power was determined by comprehensively considering the scale-free topology fit index (*R* ^2^ > 0.85) and sufficient mean connectivity. Module detection was conducted using the blockwiseModules function with parameters minModuleSize = 50 and mergeCutHeight = 0.15. Module-trait correlations were calculated using Pearson correlation coefficients. For modules significantly correlated with immune scores, gene significance (GS) and module membership (MM) were calculated to validate the reliable association between modules and traits.

Two independent gene sets were integrated for cross-screening: differentially expressed genes between tumors and normal tissues in the TCGA cohort (differential analysis using the limma package, significance threshold of adjusted *p* < 0.05) and module genes significantly correlated with immune scores obtained from WGCNA analysis. Venn diagrams were used to display the overlap between the two gene sets, with intersecting genes serving as the candidate set for potential immune-related prognostic markers.

### 2.4 Machine learning prognostic model construction

To establish a robust prognostic prediction model, multiple machine learning algorithms and their combinations were used for systematic evaluation using the candidate genes. These algorithms included: Random Survival Forest (RSF), Elastic Net (Enet), Least Absolute Shrinkage and Selection Operator (LASSO), Cox boosting algorithm (CoxBoost), Stepwise Cox regression (StepCox), Ridge regression (Ridge), partial least squares Cox regression (plsRcox), Supervised Principal Components (SuperPC), Gradient Boosting Machine (GBM), and survival support vector machine (survival-SVM). Various ensemble strategies were constructed by combining different algorithms to comprehensively assess model performance (Xun et al., 2024; Asghar et al., 2024).

For the RSF algorithm, key parameters were set as follows: the number of trees (ntree) was set to 1,000, the minimum node size (nodesize) was set to 5, and the log-rank splitting rule was applied. The mtry parameter was set to the default value (square root of the number of predictor variables). For LASSO and Ridge regression, 10-fold cross-validation was used to determine the optimal regularization parameter (lambda.min). For Elastic Net, the mixing parameter α was varied from 0.1 to 0.9 in increments of 0.1. For GBM, the number of trees was optimized through 10-fold cross-validation with an initial pool of 10,000 trees, interaction depth of 3, minimum observations per node of 10, and shrinkage rate of 0.001. All random procedures were performed with a fixed seed (seed = 1,234) to ensure reproducibility.

All models were trained on the enhanced training set and evaluated on multiple independent validation sets. Model performance was quantified by C-index, with higher C-index values indicating better prediction accuracy. For algorithms requiring hyperparameter tuning, cross-validation was used to determine optimal parameters. For algorithm combination models, the first algorithm was used to screen important variables, followed by application of the second algorithm on the subset of screened variables. C-index results for all algorithm combinations were organized as heatmaps for visual comparison. Based on average C-index on validation sets, the best-performing algorithm combination was selected for final model construction.

Based on variable importance scores from the optimal algorithm, several most important genes were selected to construct the final prognostic signature. Risk scores were calculated for each patient. Based on the median risk score in the training set as the cutoff value, patients were divided into high-risk and low-risk groups. Risk grouping results were merged with clinical information for subsequent analysis.

### 2.5 Model performance validation and clinical association analysis

In the training cohort and independent validation cohorts, the Kaplan-Meier method was used to plot survival curves for high and low-risk groups (survminer package, ggsurvplot function). Hazard ratios (HR) and 95% confidence intervals (CI) from Cox proportional hazards regression were calculated (Sjölander, 2020). Time-dependent receiver operating characteristic (ROC) curve analysis used the timeROC package to evaluate model prediction accuracy at different time points.

The relationship between risk scores and major clinicopathological features was assessed, including T stage, N stage, M stage, clinical stage, sex, and age. Violin plots combined with box plots were used to display the distribution of risk scores in different clinical subgroups. Pie charts were plotted to compare differences in the distribution of clinicopathological features between high and low-risk groups.

To evaluate the robustness of the risk score model in different clinical subgroups, stratified survival analysis was performed. Patients were stratified by age and clinical stage. Within each subgroup, patients were divided into high and low-risk groups based on the median risk score, and Kaplan-Meier survival curves were plotted. The pheatmap package was used to plot expression heatmaps of prognostic signature genes across all patients.

### 2.6 Independent prognostic value assessment and nomogram construction

To evaluate the independent prognostic value of risk scores, univariate and multivariate Cox proportional hazards regression analyses were performed. Variables included in univariate Cox regression analysis were age, sex, T stage, N stage, M stage, clinical stage, and risk score. Multivariate Cox regression analysis simultaneously included all variables to evaluate the independent prognostic significance of risk scores after adjusting for other clinicopathological factors. Forest plots were used to visualize univariate and multivariate analysis results, displaying HR, 95% CI, and *p*-values for each variable.

A comprehensive prognostic nomogram model integrating clinicopathological parameters and risk scores was constructed (rms package). The nomogram could quantify individual patient survival probabilities by accumulating scores for each parameter. Nomogram calibration was assessed through calibration curves, comparing nomogram-predicted survival probabilities with actually observed survival probabilities. The calibrate function was used for Bootstrap resampling to calculate calibration curves for survival rates at different time points.

Decision curve analysis (DCA) was used to evaluate the net benefit of the nomogram model in clinical decision-making. The rmda package was used to compare the net benefit of the nomogram model with single clinical variables at different threshold probabilities. The pec package was used to calculate time-dependent C-index for the nomogram model and each single variable. Time points were set for multiple years, and the nomogram model’s C-index should be higher than that of single variables at most time points.

### 2.7 Functional enrichment analysis

Genome-wide expression differential analysis was performed between high and low-risk groups. The limma package was used to calculate log2 fold change (logFC) for each gene (Ritchie et al., 2015). Genes were ranked in descending order by logFC to construct a pre-ranked gene list. The GSEA function in the clusterProfiler package was used to perform gene set enrichment analysis based on standard gene set databases. Significantly enriched pathways were defined as adjusted *p*-value (false discovery rate, FDR) <0.05. Normalized enrichment score (NES) indicated the group in which the pathway was enriched.

The GSVA package was used to perform GSVA on standard gene sets. The GSVA method employed the ssGSEA algorithm to generate enrichment scores for each pathway in each sample. Differential analysis (limma package) was performed on GSVA scores between high and low-risk groups to identify significantly different pathways between the two groups. Spearman correlation coefficients between each pathway’s GSVA score and risk score were calculated, and correlation heatmaps were plotted to display the association patterns between pathways and risk scores. For each pathway, patients were divided into high and low expression groups based on the median GSVA score for survival analysis. This was done to identify pathways significantly associated with prognosis and to perform Cox regression analysis.

### 2.8 Immune microenvironment analysis

Immune-related gene lists were collected from the ImmPort database and InnateDB database. Human immune-related genes from InnateDB and gene sets from the ImmPort database were merged, and duplicates were removed to construct immune-related pathway gene sets. The ssGSEA method was used to calculate enrichment scores for each TCGA-COAD sample in these immune pathways. Differential analysis was performed on immune pathway scores between high and low-risk groups, with significantly different pathways being annotated. Hierarchical clustering was used to cluster samples and pathways, and immune pathway enrichment heatmaps were plotted.

The CIBERSORT algorithm was used to estimate the relative abundance of 22 immune cell subtypes in each tumor sample. CIBERSORT deconvoluted the mixed gene expression profile of tumor tissue into proportions of different immune cell types based on the LM22 signature gene set (Chen et al., 2018). Only high-quality samples with *p* < 0.05 were retained for analysis. Abundance differences in each immune cell type between high and low-risk groups were compared using violin plots combined with box plots for visualization.

Expression data for prognostic signature genes were extracted, and Spearman correlation coefficients between these genes and immune cell abundance were calculated to generate gene-immune cell correlation matrices and corresponding *p*-value matrices. The corrplot package was used to plot correlation heatmaps, with significance levels annotated with asterisks (*). Spearman correlation coefficients between immune cell abundance and risk scores were calculated, and scatter plots and dot plots were used to display significantly correlated immune cell types.

Based on published immune cell marker gene sets, the ssGSEA method was used to calculate enrichment scores for 28 immune cell subtypes, including multiple T cell subsets, dendritic cell subsets, natural killer cells, and other immune cell types. Score differences in these immune cell subtypes between high and low-risk groups were compared using box plots for visualization.

### 2.9 Drug sensitivity analysis

The pRRophetic package was used to predict each patient’s sensitivity to commonly used anti-tumor drugs (Geeleher et al., 2014). This method was based on drug response data from the Cancer Genome Project and predicted half-maximal inhibitory concentration (IC50 values) through ridge regression models. Lower IC50 values indicated higher drug sensitivity. Multiple representative anti-tumor drugs were selected for analysis, including small molecule targeted drugs and conventional chemotherapy drugs.

Differences in IC50 value distributions for each drug between high and low-risk groups were compared using box plots for visualization. All drug sensitivity analysis results were summarized in tables, including statistical information and median IC50 values by group, sorted by *p*-value, and separately saved for all results and only significantly different drugs.

### 2.10 Key gene functional Experimental Validation

The human colon cancer cell line HCT116 and normal colon epithelial cell line used in this study were purchased from the Cell Bank of the Chinese Academy of Sciences and cultured in respective complete culture media containing 10% fetal bovine serum (FBS, Gibco) and 1% penicillin-streptomycin. All cells were cultured in a 37 °C, 5% CO_2_ incubator, and logarithmic growth phase cells were used for experiments.

Cultured cells were washed with phosphate-buffered saline (PBS), then total protein was extracted by adding radioimmunoprecipitation assay (RIPA) lysis buffer (Beyotime) containing protease inhibitors (1:100, Roche) and phosphatase inhibitors (1:100, Roche). Supernatants were collected after centrifugation at 4 °C, 12,000 rpm for 15 min, and protein concentration was measured using bicinchoninic acid (BCA) assay kit (Thermo Fisher). A total of 30 μg of total protein was separated by sodium dodecyl sulfate-polyacrylamide gel electrophoresis (SDS-PAGE) and transferred to polyvinylidene fluoride (PVDF) membranes (Millipore). Membranes were blocked with 5% non-fat milk at room temperature for 2 h, then incubated overnight at 4 °C with primary antibodies against FCGR2A (1:1,000 dilution, Elabscience), β-Actin (1:5,000 dilution, Elabscience), and β-Tubulin (1:5,000 dilution, Elabscience). The next day, membranes were incubated with horseradish peroxidase (HRP)-labeled secondary antibodies (1:5,000 dilution) at room temperature for 2 h, developed with enhanced chemiluminescence (ECL) chemiluminescent reagent kit (Millipore), images were captured using a chemiluminescent imaging system (Bio-Rad), and grayscale quantitative analysis was performed using ImageJ software.

Overexpression vectors were designed and synthesized based on target gene sequences. The target gene coding sequence (CDS) was cloned into the lentiviral vector pLVX-IRES-Puro (Clontech), and an empty vector was constructed as a negative control. Vector plasmids were co-transfected with packaging plasmid psPAX2 and envelope plasmid pMD2.G into HEK293T cells to package lentivirus. Virus-containing supernatants were collected 48 and 72 h after transfection. HCT116 cells were seeded in six-well plates at a density of 5 × 10^4^ cells/well, and cells were infected with viral supernatant containing 8 μg/mL polybrene (Sigma), with multiplicity of infection (MOI) value set to 10. Stably transfected cell lines were selected with 2 μg/mL puromycin (Sigma) 48 h after infection for 7–10 days, and target protein expression levels were verified by Western blot.

Cell Counting Kit-8 (CCK-8) assay kit (Dojindo) was used to detect cell proliferation ability. Stably transfected cells were seeded in 96-well plates at a density of 2 × 10^3^ cells/well, with five replicate wells per group. At 0, 24, 48, and 72 h after seeding, 10 μL of CCK-8 solution was added to each well. After incubation at 37 °C for 2 h, absorbance at 450 nm wavelength was measured using a microplate reader (BioTek). For colony formation assay, cells were seeded in six-well plates at a density of 500 cells/well, with three replicate wells per group. After culturing under normal culture conditions for 10–14 days, cells were fixed with 4% paraformaldehyde for 15 min and stained with 0.1% crystal violet for 20 min. Colonies containing more than 50 cells were counted using ImageJ software.

For wound healing assay, stably transfected cells were seeded in six-well plates at a density of 5 × 10^5^ cells/well. When cells reached 90% confluence, a sterile 200 μL pipette tip was used to create a straight scratch in the cell monolayer. Cells were washed three times with PBS to remove detached cells, then cultured in serum-free medium. Wound closure was photographed at 0, 24, and 48 h under a microscope (×100 magnification), and wound healing rate was calculated using ImageJ software.

Cell migration and invasion abilities were detected using 24-well Transwell chambers (8 μm pore size, Corning). For migration assay, 5 × 10^4^ cells starved for 24 h were seeded in the upper chamber with 200 μL serum-free medium in the upper chamber and 600 μL medium containing 20% FBS in the lower chamber. After culturing for 24 h, cells were fixed and stained. For invasion assay, 50 μL of diluted Matrigel matrix gel (1:8 dilution, BD Biosciences) was pre-coated in the upper Transwell chamber and polymerized at 37 °C for 1 h before seeding 1 × 10^5^ cells. After culturing for 48 h, cells were fixed and stained. Cotton swabs were used to remove unmigrated/uninvaded cells from the upper chamber. Cells were fixed with 4% paraformaldehyde for 20 min and stained with 0.1% crystal violet for 15 min. Transmembrane cells were counted in five randomly selected fields of view (×200 magnification) under a microscope. All experiments included three biological replicates.

### 2.11 Statistical analysis

All statistical analyses and data visualization were completed using R software (version 4.2.0) and its related extension packages. Continuous variable data were expressed as mean ± standard deviation (SD), and categorical variables were expressed as frequencies and percentages. Continuous variables between two groups were compared using Student’s t-test (normally distributed data) or Wilcoxon rank-sum test (non-normally distributed data). Multiple group comparisons used one-way analysis of variance (ANOVA) or Kruskal–Wallis H test. Categorical variables were compared using chi-square test or Fisher’s exact test. Survival analysis used the Kaplan-Meier method to plot survival curves, and log-rank test was used to compare survival differences between groups. Univariate and multivariate Cox proportional hazards regression models were used to evaluate prognostic factors, with results expressed as HR and 95% CI. Predictive performance of prognostic models was evaluated by C-index and time-dependent area under the ROC curve (AUC). Gene expression correlation analysis used Pearson correlation coefficient (normal distribution) or Spearman rank correlation coefficient (non-normal distribution). Significance thresholds for functional enrichment analysis were set at adjusted *p* value (FDR or adjusted *p* value) < 0.05. All hypothesis tests were two-sided, with *p* < 0.05 considered statistically significant. Significance levels were annotated as *p < 0.05, **p < 0.01, ***p < 0.001, ****p < 0.0001.

## 3 Results

**FIGURE 1:**
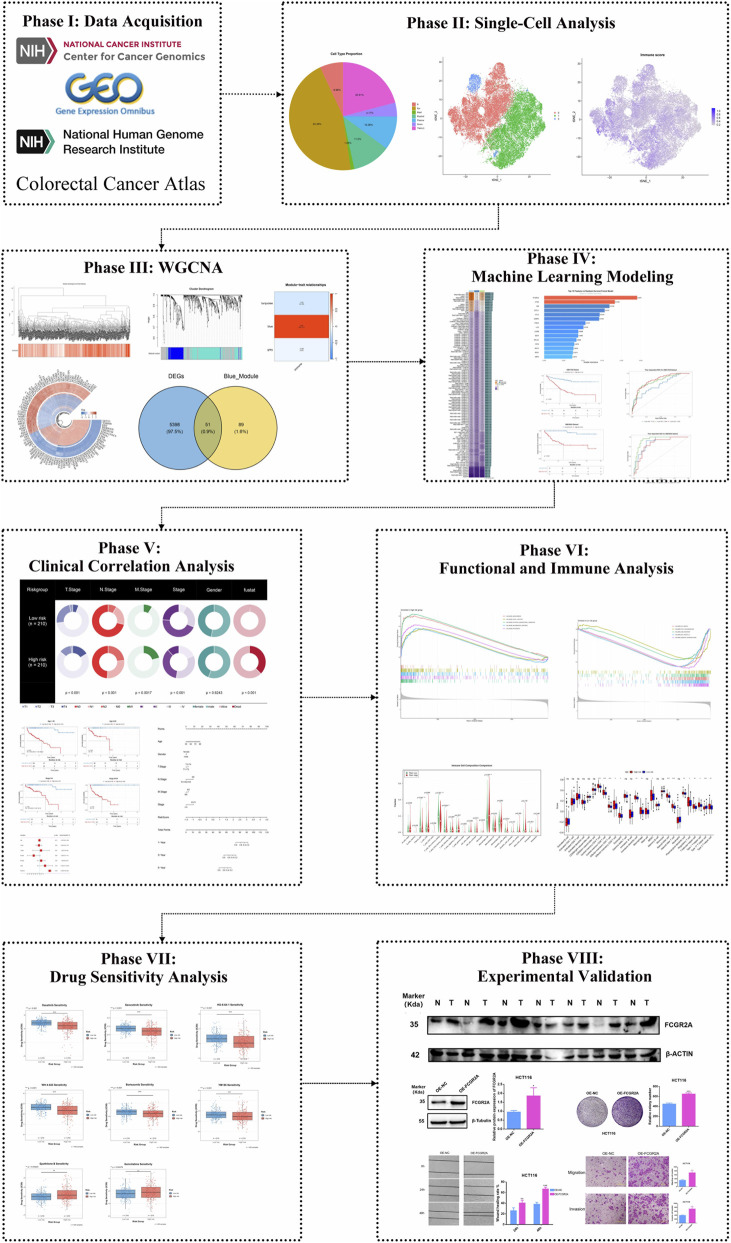
Research flowchart.

The process of this study is as follows (Figure 1).

### 3.1 Single-cell RNA sequencing reveals distinct immune cell populations in colon cancer and identifies T cell heterogeneity based on ICD score

**FIGURE 2:**
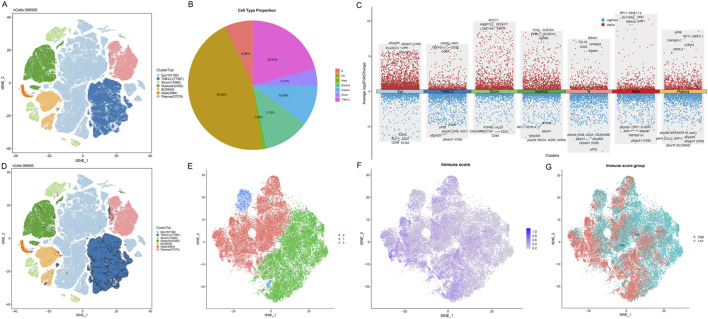
Single-cell RNA sequencing analysis identifies T cell heterogeneity based on ICD score in the colon cancer microenvironment. **(A)** t-SNE visualization plot of seven major cell types identified in the colon cancer tumor microenvironment, with each color representing a different cell population. Due to marker gene similarity, T cells, NK cells, and ILC were merged into the TNKILC population. **(B)** Pie chart showing the proportion of each cell type. **(C)** Dot plot showing the expression patterns of classical marker genes in different cell types. The size of the dots represents the percentage of cells expressing the gene, and the color intensity indicates the average expression level. **(D)** t-SNE plot highlighting the spatial distribution of the TNKILC cell population. **(E)** t-SNE plot of re-clustered TNKILC cells after extraction. **(F)** Feature plot showing the distribution of ICD scores in T cell subpopulations, with color gradient indicating score intensity from low (gray) to high (purple). **(G)** T cells classified into high ICD score group (High, red) and low ICD score group (Low, cyan) based on the median ICD score.

To systematically explore the heterogeneity of immune cells in the colon cancer tumor microenvironment, we performed an in-depth analysis of single-cell RNA sequencing data from the Colon Cancer Atlas Database. After rigorous quality control, we retained high-quality single-cell transcriptome data for subsequent analysis. Using t-SNE dimensionality reduction and unsupervised clustering analysis, we successfully identified seven major cell type populations in the colon cancer tumor microenvironment (Figure 2A). Since T cells, natural killer (NK) cells, and innate lymphoid cells (ILC) express similar marker genes, we merged these three cell types into the TNKILC population for analysis. Cell type proportion analysis showed that epithelial cells (Epi) constituted the largest cell population, accounting for 45.36% of total cells, followed by TNKILC (20.91%), myeloid cells (Myeloid, 11.5%), plasma cells (Plasma, 10.06%), B cells (6.96%), stromal cells (Strom, 4.17%), and mast cells (Mast, 1.05%) (Figure 2B). This finding revealed the complex cellular composition of the colon cancer tumor microenvironment, in which immune cell populations occupied a considerable proportion, suggesting that the immune microenvironment may play an important role in the development and progression of colon cancer.

Further refined analysis revealed the molecular characteristics and marker gene expression patterns of major cell types (Figure 2C). We observed that different cell subpopulations exhibited unique gene expression profiles: TNKILC cells highly expressed lymphocyte markers and cytotoxicity-related genes such as CD3E, CD247, CD8A, GZMM, and PRF1; epithelial cells were enriched in epithelium-specific marker genes such as PLA2G10 and LIPH; myeloid cells significantly expressed myeloid markers such as FCN1, FPR3, CLEC5A, and FCGR1A; B cells specifically expressed B cell marker genes such as CD22, TCL1A, MS4A1, and BANK1. These results validated the accuracy of our cell type annotation.

Given the importance of the TNKILC cell population in tumor immune response, we conducted a more in-depth analysis of this population. We first highlighted the spatial distribution of the TNKILC cell population in the t-SNE plot of all cells (Figure 2D), and subsequently extracted TNKILC cells for re-clustering analysis. After re-clustering, to focus on the functional characteristics of T cells, we excluded non-T cell subpopulations based on marker gene expression patterns, and finally retained three pure T cell subpopulations (cluster 0, 1, and 3) for subsequent analysis (Figure 2E).

To evaluate the activity of ICD-related signaling pathways in T cell subpopulations, we calculated the ICD score for each T cell using the ssGSEA algorithm based on ICD-related gene sets. The t-SNE visualization results showed that ICD scores exhibited a distinct spatial gradient distribution in T cell subpopulations (Figure 2F), suggesting that different T cell subpopulations may have differential immunogenicity recognition and response capabilities. Based on the median ICD score, we divided T cells into two functional subpopulations: high ICD score group (High) and low ICD score group (Low) (Figure 2G). Notably, T cells with high ICD scores and T cells with low ICD scores exhibited an obvious separation trend in spatial distribution, suggesting that these two T cell subpopulations may have different functional states and anti-tumor activities.

### 3.2 Identification of key gene modules associated with ICD based on WGCNA

**FIGURE 3:**
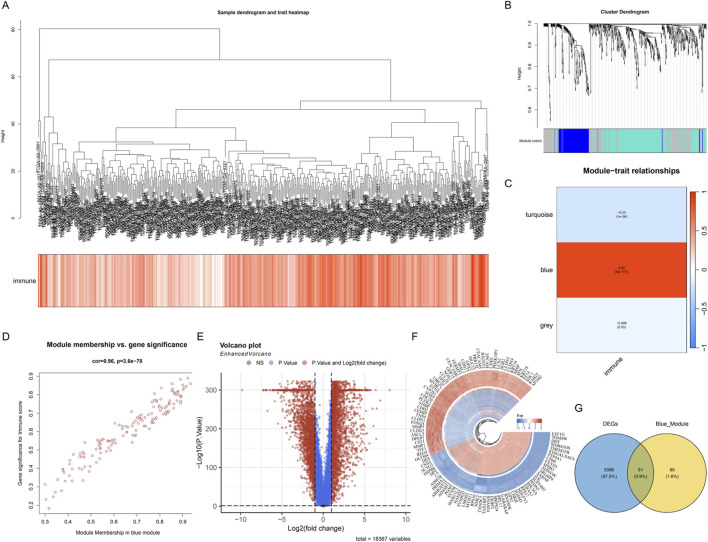
WGCNA identifies key gene modules associated with immunity in colon cancer. **(A)** Sample clustering dendrogram and immune score heatmap, showing the clustering relationships of samples from the TCGA-COAD cohort and the heterogeneity of their immune infiltration characteristics. **(B)** Gene clustering dendrogram, classifying genes with similar expression patterns into different co-expression modules through hierarchical clustering, with each color representing an independent gene module. **(C)** Module-trait relationship heatmap, showing the correlation between module eigengenes and immune scores. The values and color intensities in the heatmap represent correlation coefficients and statistical significance, respectively, with the blue module showing a strong positive correlation with immune scores. **(D)** Scatter plot of gene membership *versus* gene significance in the blue module. **(E)** Volcano plot showing the distribution of differentially expressed genes between tumor tissues and normal tissues in the TCGA cohort. Red dots represent significantly upregulated genes, blue dots represent significantly downregulated genes, and gray dots represent genes without significant differences. **(F)** Heatmap showing the expression patterns of the top genes with the most significant differences between tumor samples and normal samples, with color intensity indicating the level of gene expression. **(G)** Venn diagram showing the intersection of differentially expressed genes (DEGs) between tumor and normal tissues in the TCGA cohort and WGCNA blue module genes.

To systematically identify gene modules associated with the colon cancer immune microenvironment, we performed WGCNA on transcriptome data from the TCGA-COAD cohort. First, we constructed a sample clustering dendrogram and combined it with a heatmap of ICD scores to display the correlation between samples and immune infiltration characteristics (Figure 3A). The sample clustering results showed that different samples exhibited obvious heterogeneity at the transcriptome level, and immune scores varied significantly among samples. By setting appropriate soft threshold parameters, we performed hierarchical clustering analysis on genes and successfully identified multiple co-expression gene modules with different expression patterns (Figure 3B). Soft-threshold analysis identified β = 5 (scale-free *R* ^2^ = 0.85) as optimal. WGCNA detected three modules, among which the blue module showed the strongest correlation with immune score (Figure 3C, *r* = 0.92, *p* < 0.001). Each module was marked with a different color, representing a collection of genes highly correlated in expression patterns. These gene modules may represent different biological functional pathways or cellular processes. To evaluate the association between gene modules and immune infiltration characteristics, we calculated the correlation between module eigengenes and immune scores (Figure 3C). The module-trait relationship heatmap clearly showed the strength and direction of associations between different modules and immune features. Among them, the blue module exhibited a significant positive correlation with immune scores (*r* = 0.92, *p* < 0.001), while the turquoise module and grey module showed weaker or negative correlation trends.

Further analysis focused on the blue module with the strongest ICD correlation. We plotted a scatter plot of gene membership *versus* gene significance for genes in this module (Figure 3D), and the results showed a highly positive correlation between the two, validating the reliable association between this module and ICD features. To comprehensively screen key genes with clinical significance, we performed differential expression analysis between tumor tissue and normal tissue samples in the TCGA cohort. The volcano plot clearly showed the distribution pattern of differentially expressed genes (Figure 3E), revealing many genes significantly differentially expressed between tumor and normal tissues. Subsequently, we visualized the top genes with the most significant differences using a heatmap (Figure 3F), showing the expression pattern differences of these key genes between tumor samples and normal samples. These differentially expressed genes may play important roles in the development and progression of colon cancer. Finally, we integrated two independent gene sets for cross-screening: differentially expressed genes (DEGs) between tumor and normal tissues in the TCGA cohort and blue module genes (Blue_Module) from WGCNA analysis. The Venn diagram showed the overlap between these two gene sets (Figure 3G), successfully identifying 55 candidate genes that simultaneously met both analysis conditions. These intersection genes are both key members of the immune-related co-expression network and show significant differential expression in tumor tissues, and are therefore considered potential immune-related prognostic markers, providing an important candidate gene set for subsequent construction of prognostic models.

### 3.3 Construction and validation of a prognostic risk model based on ICD-related genes

**FIGURE 4:**
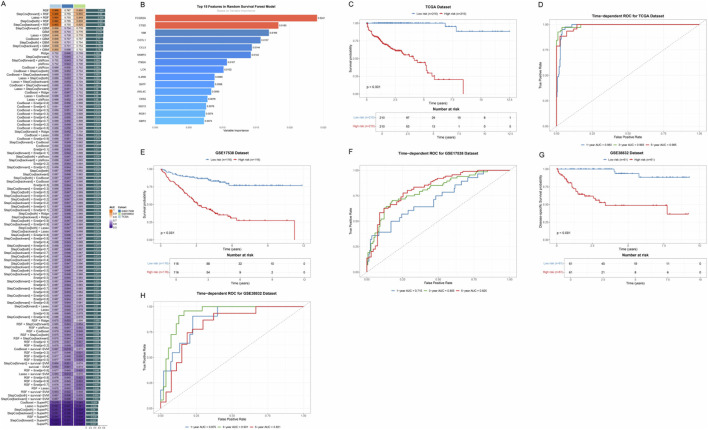
Prognostic risk model based on ICD-related genes. **(A)** Performance comparison heatmap of 117 machine learning algorithm combinations. Each square represents an algorithm combination, and the color depth indicates the C-index value of that combination for evaluating prediction accuracy. **(B)** Bar chart showing the 15 key immune-related prognostic genes screened by the RSF model and their relative importance. **(C)** Kaplan-Meier survival curves based on risk scores in the TCGA training cohort. The blue curve represents the low-risk group, and the red curve represents the high-risk group. **(D)** Time-dependent ROC curves in the TCGA cohort, evaluating the prediction accuracy of the model at different time points. **(E)** Kaplan-Meier survival curves in the GSE17536 validation cohort. **(F)** Time-dependent ROC curves in the GSE17536 cohort. **(G)** Kaplan-Meier survival curves in the GSE38832 validation cohort. **(H)** Time-dependent ROC curves in the GSE38832 cohort.

To establish a robust prognostic prediction model, we first systematically evaluated the candidate genes screened in the previous stage using various machine learning algorithms. By integrating 117 different algorithm combinations, including RSF, Enet, LASSO, CoxBoost, StepCox, Ridge, and SuperPC, among other modeling strategies, we comprehensively compared the predictive performance of each algorithm combination (Figure 4A). The heatmap showed the performance of different algorithm combinations in terms of C-index, with color depth representing the level of prediction accuracy. Through systematic performance evaluation, the algorithm combination with the best performance (RSF) was ultimately selected for subsequent model construction.

Based on RSF, we successfully screened 15 key immune-related prognostic genes and constructed a multi-gene risk score model (Figure 4B). These genes included FCGR2A, CTSD, VIM, COTL1, CCL5, VAMP2, ITM2A, LCK, IL2RB, BATF, ARL4C, CD52, ISG15, RGS1, and GBP2. The bar chart showed the relative importance of each gene, with genes such as FCGR2A and CTSD showing higher weights.

To validate the predictive performance of this prognostic model, we performed survival analysis in the TCGA training cohort. Based on the risk scores calculated by the model, we divided patients into high-risk and low-risk groups. The Kaplan-Meier survival curve showed that there was a significant difference in OS between the two groups (*p* < 0.001), with patients in the high-risk group having significantly worse prognosis than those in the low-risk group (Figure 4C). Time-dependent ROC curves further evaluated the prediction accuracy of the model at different time points (Figure 4D), and the results showed that the model maintained high predictive ability at multiple time nodes. To verify the stability and generalizability of the model, we validated the model performance in two independent external validation cohorts, GSE17538 and GSE38832. Survival analysis in both cohorts again validated the prognostic value of the risk score (*p* < 0.001) (Figures 4E–G), and time-dependent ROC curves showed that the AUC values of the model remained at high levels at different time points (Figures 4F–H).

### 3.4 Association analysis of the risk score model with clinicopathological features

**FIGURE 5:**
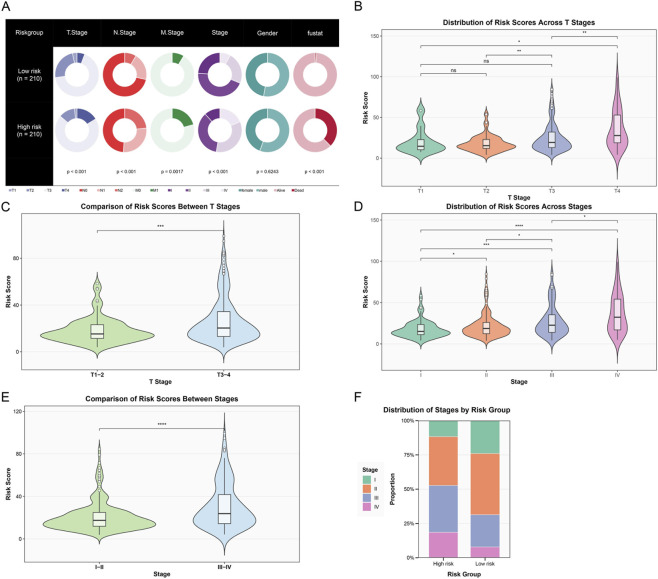
Association analysis of the risk score model with clinicopathological features. **(A)** Pie charts showing the distribution of patients in the low-risk group (*n* = 210) and high-risk group (*n* = 210) across different clinicopathological features, including T stage, N stage, M stage, overall Stage, Gender, and prognostic status. **(B)** Violin plot showing the distribution of risk scores in different T stages (T1–T4). Statistical annotations show significant differences between different stages. **(C)** Violin plot comparing the distribution of risk scores between early T stage (T1–2) and late T stage (T3-4) patients, with highly significant differences between the two groups. **(D)** Violin plot showing the distribution of risk scores in different overall stages (Stage I–IV), with risk scores gradually increasing with stage progression, showing significant differences between late and early stages. **(E)** Violin plot comparing the distribution of risk scores between early (Stage I–II) and late (Stage III–IV) patients, with risk scores in the late group significantly higher than in the early group. **(F)** Stacked bar chart showing the proportion distribution of patients at each clinical stage (Stage I–IV) in the high-risk and low-risk groups. (ns, not significant; *p < 0.05; **p < 0.01; ***p < 0.001; ****p < 0.0001).

To deeply explore the relationship between the constructed immune-related prognostic risk model and clinicopathological features of colon cancer, we systematically analyzed the distribution patterns of risk scores in different clinical subgroups and their clinical significance. First, we comprehensively displayed the distribution differences of patients in the low-risk and high-risk groups across multiple key clinicopathological features through pie charts (Figure 5A). The analysis covered multiple dimensions including T stage, N stage, M stage, overall Stage, Gender, and fustat (survival status). The results showed that the two groups exhibited statistical differences in T stage, lymph node metastasis, M stage, and overall stage (*p* < 0.05). Gender distribution showed no significant difference between the two groups (*p* = 0.6243), while prognostic status showed highly significant differences between the two groups (*p* < 0.001), directly validating the prognostic prediction capability of the risk score model. Given that T stage and overall Stage are the two most important independent prognostic factors for colon cancer and hold a central position in clinical decision-making, we focused on the relationship between risk scores and these two key clinical staging systems in subsequent analyses.

Further analysis of the distribution characteristics of risk scores in different T stages (Figure 5B) showed that the violin plot clearly demonstrated a progressive increase in risk scores with the advancement of T stage (from T1 to T4). When we simplified T stage into early (T1–2) and late (T3–4) groups for comparison (Figure 5C), the risk scores in the late T stage group were significantly higher than those in the early group (*p* < 0.001), further confirming the strong correlation between risk scores and tumor local invasiveness. In the analysis of overall tumor staging (Figure 5D), risk scores showed a continuous increase with stage progression (from Stage I to Stage IV). Similarly, when we grouped patients by early (I–II) and late (III–IV) stages for comparison (Figure 5E), the risk scores of late-stage patients were significantly higher than those of early-stage patients (*p* < 0.0001). Finally, a stacked bar chart showed the proportion distribution of patients at each clinical stage in different risk groups (Figure 5F). It could be clearly observed that the proportion of late-stage patients (Stage III–IV) in the high-risk group was significantly higher than in the low-risk group, while the low-risk group was enriched with more early-stage patients (Stage I–II). In summary, our constructed immune-related risk score model was significantly associated with multiple important clinicopathological features, particularly closely related to tumor T stage and overall stage. These findings not only validated the clinical relevance of the model but also provided strong evidence for its application in clinical practice.

### 3.5 Validation of the prognostic prediction capability of the risk score model in different clinical subgroups

**FIGURE 6:**
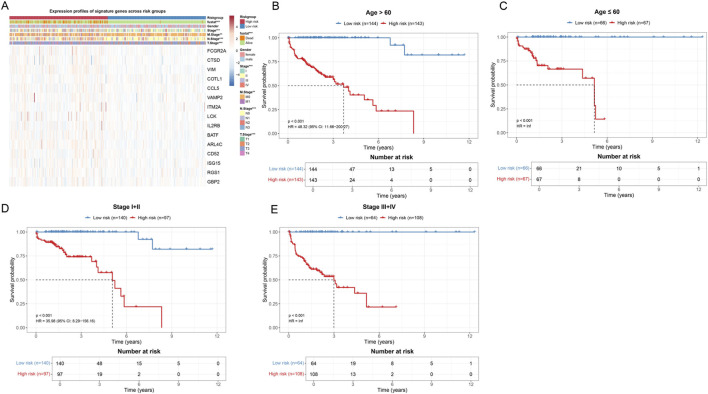
Validation of the prognostic prediction capability of the risk score model in different clinical subgroups. **(A)** Heatmap showing the expression profiles of 15 signature genes in all patient samples. The annotation bars at the top identify a series of clinicopathological features. In the heatmap, red indicates high expression and blue indicates low expression. **(B)** Kaplan-Meier survival curves for patients older than 60 years. The blue curve represents the low-risk group, and the red curve represents the high-risk group. The risk table below shows the number of surviving patients at different time points. **(C)** Kaplan-Meier survival curves for patients aged less than or equal to 60 years. **(D)** Kaplan-Meier survival curves for early-stage patients (Stage I–II). **(E)** Kaplan-Meier survival curves for late-stage patients (Stage III–IV).

To further validate the robustness and generalizability of the constructed immune-related risk score model in different clinical subgroups, we performed stratified survival analysis on patients. This strategy aimed to evaluate whether the risk score could maintain its prognostic prediction capability in patient populations with different clinical characteristics, thereby providing more comprehensive evidence support for the clinical application of the model. First, the heatmap showed the expression patterns of the 15 signature genes in patients from the high-risk and low-risk groups (Figure 6A). The annotation bars at the top clearly identified multiple clinicopathological parameters including risk group, fustat, Stage, Gender, M stage, N stage, and T stage. The heatmap showed obvious gene expression pattern differences between the high-risk and low-risk groups. Notably, all 15 signature genes (FCGR2A, CTSD, VIM, COTL1, CCL5, VAMP2, ITM2A, LCK, IL2RB, BATF, ARL4C, CD52, ISG15, RGS1, GBP2) exhibited high expression characteristics in the high-risk group. This consistent high expression pattern suggested that these genes may synergistically act on immune regulation in the tumor microenvironment, jointly promoting tumor progression and poor prognosis. This finding also validated the rationality of the risk score formula constructed based on Cox regression coefficients, in which all gene regression coefficients were positive values, reflecting their positive correlation with poor prognosis.

In age stratification analysis, we divided patients into an age greater than 60 years group (Figure 6B) and an age less than or equal to 60 years group (Figure 6C). For patients older than 60 years (*n* = 287), the Kaplan-Meier survival curve showed a significant survival difference between the low-risk group (*n* = 144) and high-risk group (*n* = 143) (*p* < 0.001, HR = 48.32, 95% CI: 11.66–200.27). The median survival time of high-risk group patients was significantly shortened. In patients aged less than or equal to 60 years (*n* = 133), the risk score model still showed significant prognostic discrimination ability (*p* < 0.001, HR = inf), with the survival curve of the low-risk group patients (*n* = 66) significantly better than that of the high-risk group (*n* = 67).

Furthermore, we performed stratified analysis according to clinical stage. For early-stage patients (Stage I–II, *n* = 237) (Figure 6D), the low-risk group (*n* = 140) and high-risk group (*n* = 97) also showed significant survival differences (*p* < 0.001, HR = 35.98, 95% CI: 8.29–156.16). Notably, among early-stage patients who are traditionally considered to have better prognosis, the risk score model could still identify a subset of high-risk patients with poor prognosis, which has important implications for guiding individualized treatment decisions for early-stage patients. In late-stage patients (Stage III–IV, *n* = 172) (Figure 6E), the risk score model likewise maintained strong prognostic prediction capability (*p* < 0.001, HR = inf).

In summary, through systematic validation in different age and clinical stage subgroups, we confirmed that the constructed immune-related risk score model has good robustness and generalizability. The model can not only distinguish patient populations with different prognoses in the overall cohort but also maintain its predictive ability in various clinical subgroups, which lays a solid foundation for its widespread application in clinical practice.

### 3.6 Construction and validation of a clinical prognostic prediction model

**FIGURE 7:**
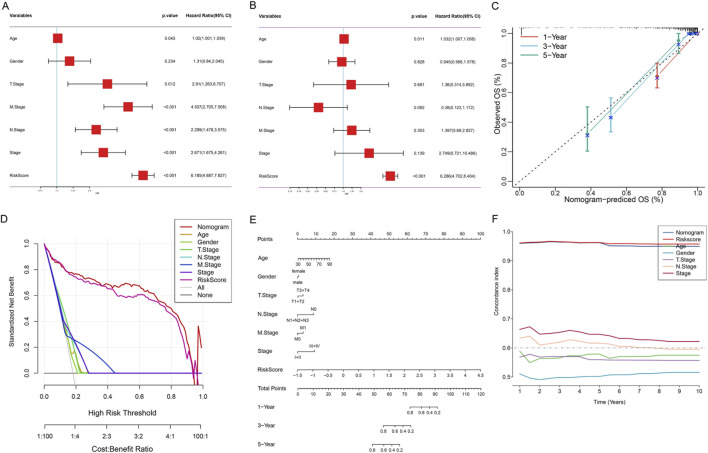
Construction and Validation of the Nomogram Model for OS Prediction in Colon Cancer Patients. **(A)** Forest plot of univariate Cox regression analysis. **(B)** Forest plot of multivariate Cox regression analysis. **(C)** Calibration curves of the nomogram model. The X-axis represents the OS rate predicted by the nomogram, and the Y-axis represents the actual observed OS rate. Different colored lines represent 1-year (orange), 3-year (blue), and 5-year (green) survival rate predictions. The dashed line represents the ideal prediction line (slope = 1). Error bars represent the 95% confidence interval from Bootstrap resampling. **(D)** DCA. The X-axis represents threshold probability, and the Y-axis represents standardized net benefit. The lower X-axis shows the cost-benefit ratio. **(E)** Comprehensive prognostic nomogram. The three axes at the bottom represent the predicted values of 1-year, 3-year, and 5-year OS rates, respectively. **(F)** Time-dependent C-index curves. The X-axis represents follow-up time (years), and the Y-axis represents C-index values.

To evaluate the independent prognostic value of the risk score model and construct a prediction tool suitable for clinical application, we performed systematic Cox regression analysis and constructed a nomogram model. Univariate Cox regression analysis showed that except for gender (HR = 1.31, 95% CI: 0.84–2.05, *p* = 0.234), age, T stage, M stage, N stage, clinical stage, and risk score (*p* < 0.05) were all significantly associated with OS (Figure 7A). Notably, the risk score showed the highest hazard ratio, suggesting its strong prognostic prediction capability. To comprehensively evaluate the independent prognostic value of various clinicopathological factors, we included all variables in multivariate Cox regression analysis. The results showed that after adjusting for other clinicopathological factors, age (HR = 1.03, 95% CI: 1.01–1.06, *p* = 0.011) and risk score (HR = 6.29, 95% CI: 4.70–8.40, *p* < 0.001) still maintained independent prognostic significance (Figure 7B), further confirming the value of risk score as an independent prognostic factor.

Subsequently, we integrated age, gender, T stage, N stage, M stage, clinical stage, and risk score to construct a comprehensive prognostic nomogram model (Figure 7E). This model can quantify individual patients’ 1-, 3-, and 5-year survival probabilities by accumulating the scores of various clinicopathological parameters. Calibration curve analysis showed that the nomogram performed well in predicting 1-, 3-, and 5-year OS rates, with predicted values highly consistent with actual observed values (Figure 7C), indicating excellent prediction accuracy of the model. DCA results showed that within most threshold probability ranges, the net benefit of the nomogram model was significantly higher than that of all single clinical variables, including age, gender, TNM stage, clinical stage, and risk score (Figure 7D). This indicated that the nomogram model has higher practical value in clinical decision-making and can provide more accurate prognostic assessment for patients. Time-dependent C-index analysis showed that the nomogram model maintained stable and highest C-index values (approximately 0.65–0.70) during the 1–10 years follow-up period, significantly better than the single risk score (approximately 0.60–0.65) and other clinical variables (Figure 7F). Especially in long-term follow-up, the prediction performance of the nomogram model remained stable, while the predictive ability of some clinical variables decreased over time, further confirming the superiority and robustness of the integrated model.

### 3.7 Functional enrichment analysis of risk groups

**FIGURE 8:**
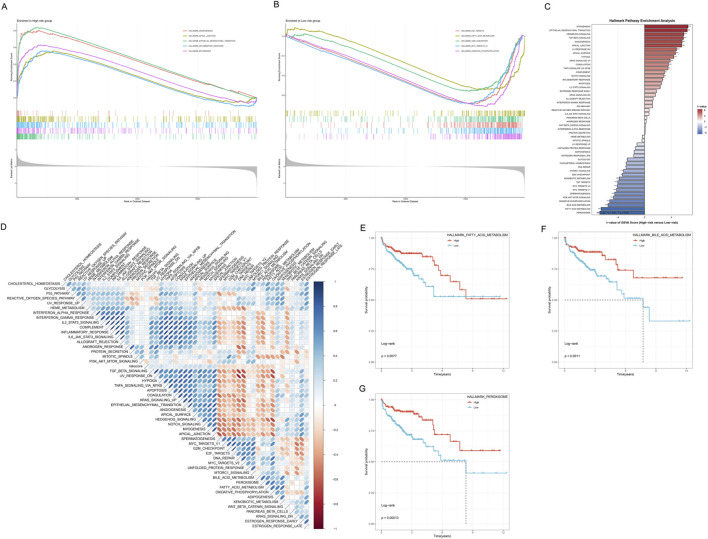
Functional Enrichment Analysis of High-risk and Low-risk Groups. **(A)** GSEA enrichment curves for the high-risk group. **(B)** GSEA enrichment curves for the low-risk group. **(C)** Hallmark Pathway Enrichment Analysis bar chart. The Y-axis lists the names of enriched Hallmark gene sets, and the X-axis represents Enrichment Score (negative Log10(FDR)). The right-side bar chart (red tones) shows pathways positively enriched in the high-risk group, and the left-side bar chart (blue tones) shows pathways positively enriched in the low-risk group. **(D)** Enrichment heatmap of 50 Hallmark pathways in high-risk and low-risk groups. **(E)** Kaplan-Meier curves of HALLMARK_FATTY_ACID_METABOLISM pathway expression and OS. **(F)** Kaplan-Meier curves of HALLMARK_BILE_ACID_METABOLISM pathway expression and OS. **(G)** Kaplan-Meier curves of HALLMARK_PHAGOSOME pathway expression and OS.

To deeply understand the molecular mechanism differences between high-risk and low-risk groups, we performed comprehensive functional enrichment analysis on patients in both groups, including GSEA and pathway enrichment analysis. GSEA results showed that multiple signaling pathways related to tumor progression and invasion were significantly enriched in high-risk group patients (Figure 8A). These pathways mainly included HALLMARK_ANGIOGENESIS, HALLMARK_APICAL_JUNCTION, HALLMARK_EPITHELIAL_MESENCHYMAL_TRANSITION, HALLMARK_INFLAMMATORY_RESPONSE, and HALLMARK_MYOGENESIS. Enrichment curves showed that these pathway genes exhibited a continuous positive enrichment trend in the high-risk group, indicating that the tumor microenvironment of high-risk patients has stronger invasive characteristics and more active tumor-promoting signals. In contrast, low-risk group patients were significantly enriched in a series of metabolism and cell proliferation-related pathways (Figure 8B), including HALLMARK_E2F_TARGETS, HALLMARK_FATTY_ACID_METABOLISM, HALLMARK_G2M_CHECKPOINT, HALLMARK_MYC_TARGETS_V1, and HALLMARK_OXIDATIVE_PHOSPHORYLATION, among other basic metabolic and cell cycle regulation processes. The enrichment of these metabolic pathways suggested that low-risk group tumors may retain more normal tissue metabolic characteristics, with relatively weaker cell proliferation and invasion capabilities.

Hallmark gene set enrichment analysis further revealed the functional characteristic differences of risk groups (Figure 8C). Significantly enriched pathways in the high-risk group included EPITHELIAL_MESENCHYMAL_TRANSITION, HYPOXIA, ANGIOGENESIS, APOPTOSIS, and COMPLEMENT, among other biological processes closely related to tumor progression, while the low-risk group was enriched in metabolism-related pathways such as OXIDATIVE_PHOSPHORYLATION, FATTY_ACID_METABOLISM, and BILE_ACID_METABOLISM. This result was highly consistent with the GSEA analysis results, further validating that the risk scoring system can effectively distinguish patient subgroups with different biological characteristics. To more comprehensively understand the pathway differences of risk groups, we systematically compared the enrichment levels of 50 Hallmark gene sets in high-risk and low-risk groups (Figure 8D). This obvious contrast in pathway activity highlighted the essential differences in tumor biological behavior between the two patient groups.

To further validate the prognostic value of key pathways, we performed survival analysis on three representative Hallmark pathways. High expression of HALLMARK_FATTY_ACID_METABOLISM, HALLMARK_BILE_ACID_METABOLISM, and HALLMARK_PHAGOSOME pathways all showed significantly better OS (*p* < 0.05, Figures 8E–G).

In summary, functional enrichment analysis revealed that high-risk group patients are characterized by activation of pathways related to tumor invasion, metastasis, and angiogenesis, among other malignant phenotypes, while the low-risk group is mainly characterized by maintenance of basic metabolic pathways and cell cycle regulation. These findings provide a solid molecular biological foundation for the clinical application of the risk scoring system.

### 3.8 Immune landscape and tumor microenvironment analysis

**FIGURE 9:**
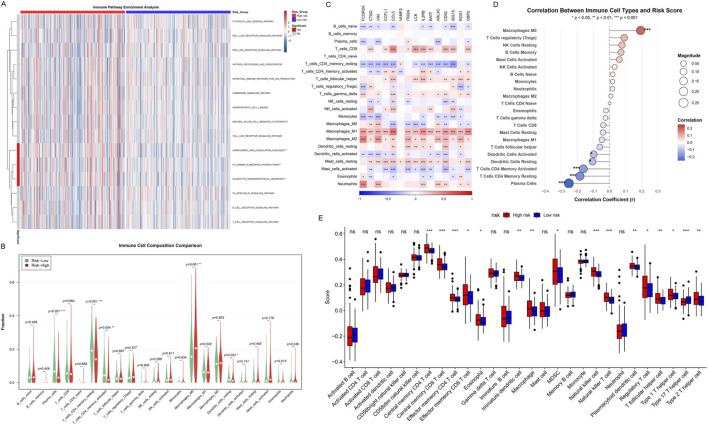
Immune Landscape and Tumor Microenvironment Analysis. **(A)** Immune pathway enrichment analysis heatmap, showing ssGSEA scores across TCGA-COAD samples. Red: high enrichment; blue: low enrichment. Top bar: risk groups (red: high-risk; blue: low-risk). Dendrograms show hierarchical clustering of samples and pathways. **(B)** Violin plots comparing the composition of 22 immune cell types. **(C)** Heatmap of the expression of 15 key immune-related genes in 22 immune cell types. Colors represent gene expression levels, with red indicating high expression and blue indicating low expression. **(D)** Scatter plots of correlation between immune cell infiltration and risk scores. **(E)** Box plots comparing the infiltration levels of 28 immune cell subtypes in high-risk and low-risk groups. Asterisks indicate significance levels (***p < 0.001, **p < 0.01, *p < 0.05).

To comprehensively analyze the relationship between the risk scoring system and the tumor immune microenvironment, we performed an in-depth analysis of the immune cell infiltration characteristics of patients in the high-risk and low-risk groups. First, we used the ssGSEA algorithm to systematically evaluate the enrichment of 15 immune-related pathways in all samples (Figure 9A). The heatmap showed that samples could be clearly clustered into two major subgroups based on immune pathway enrichment patterns, and these two subgroups highly corresponded to the high-risk and low-risk groups. High-risk group samples (red annotation) showed higher enrichment scores (red) in most immune-related pathways, while low-risk group samples (blue annotation) showed relatively lower immune pathway activity (blue). The hierarchical clustering dendrogram showed the similarity of immune characteristics among samples, further supporting the immunological differences between risk groups. This result suggested that the risk scoring system can effectively reflect the overall state of the tumor immune microenvironment.

To quantify the infiltration differences of different immune cell types in the high-risk and low-risk groups, we used multiple immune cell deconvolution algorithms to estimate the abundance of 22 immune cell subpopulations (Figure 9B). Violin plots showed that multiple immune cell types differed significantly between the two groups. Notably, the high-risk group showed trends of higher levels of Macrophages M0, Macrophages M2, and T cells regulatory (Tregs) infiltration, which are usually associated with immunosuppression and tumor progression. In contrast, the low-risk group showed higher levels of T cells CD4 memory resting, Plasma cells, and certain activated immune cells, suggesting a more effective anti-tumor immune response.

To deeply understand the molecular characteristics of different immune cell types, we analyzed the expression of 15 key immune-related genes in 22 immune cell types (Figure 9C). The heatmap showed the expression patterns of these key genes in different immune cell types, revealing obvious cell type-specific expression profiles. Notably, Macrophages M1, Macrophages M2, and T cells CD8 universally highly expressed these key immune-related genes (red areas), and significance tests showed that most genes reached statistical significance in these cell types. This finding suggested that macrophages and cytotoxic T cells are in a highly activated state in immune response, and their enhanced transcriptional activity may be closely related to tumor immune surveillance and inflammatory responses. In contrast, B cells naive, T cells CD4 memory resting, and Mast cells universally showed low expression of these key genes (blue areas), indicating that these cell types are in a relatively quiescent state or have different molecular regulatory mechanisms. This difference in expression patterns not only reflects the functional heterogeneity of different immune cell subpopulations but also provides important molecular evidence for understanding the complexity of the immune microenvironment, while revealing the intrinsic connection between the risk scoring system and immune cell functional states.

Correlation analysis further quantified the relationship between the infiltration levels of various immune cell types and risk scores (Figure 9D). Scatter plots showed that Macrophages M0 was significantly positively correlated with risk scores (*p* < 0.001). In contrast, T cells CD4 memory activated, Dendritic cells activated, Dendritic cells resting, T cells CD4 memory resting, and Plasma cells were significantly negatively correlated with risk scores (*p* < 0.05 to *p* < 0.001), suggesting that low-risk patients have stronger anti-tumor immune activity.

Finally, we comprehensively compared the infiltration levels of 28 immune cell subtypes in the high-risk and low-risk groups (Figure 9E). Box plots showed that the high-risk group (red) exhibited significantly higher infiltration levels in multiple immunosuppressive cell types, including Central memory CD4 T cell (*p* < 0.001), Central memory CD8 T cell (*p* < 0.001), Effector memory CD4 T cell (*p* < 0.001), Effector memory CD8 T cell (*p* < 0.05), Immature dendritic cell (*p* < 0.01), Macrophage (*p* < 0.01), NK cell (*p* < 0.001), NK T cell (*p* < 0.001), Plasmacytoid dendritic cell (*p* < 0.01), Treg (*p* < 0.05), T follicular helper cell (*p* < 0.01), and Type 2 T helper cell (*p* < 0.01). The low-risk group showed relatively higher but non-significantly different infiltration levels in certain cell types such as Activated dendritic cell.

Both CIBERSORT and ESTIMATE consistently identified higher immune activity in the low-risk group, supporting the robustness of our findings. In summary, immune landscape analysis indicated that the tumor microenvironment of high-risk group patients is mainly characterized by immunosuppressive cell infiltration, while the low-risk group shows a more favorable anti-tumor immune response.

### 3.9 Drug sensitivity analysis

**FIGURE 10:**
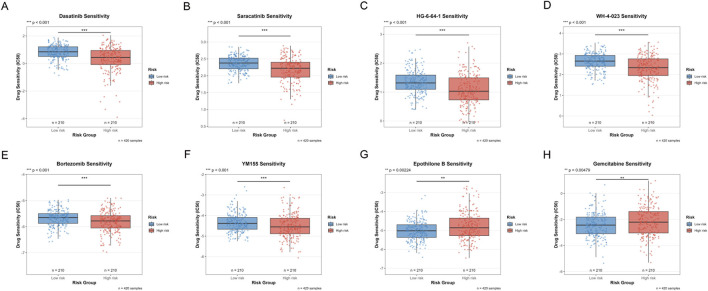
Drug Sensitivity Analysis Between High and Low Risk Groups. **(A–H)** Box plots comparing the sensitivity of eight anti-tumor drugs in high-risk and low-risk groups. The Y-axis represents Drug Sensitivity (IC50), the X-axis shows Risk Group, the blue box plot represents the Low-risk group (*n* = 210), and the red box plot represents the High-risk group (*n* = 210), with a total sample size of n = 420. (***p < 0.001, **p < 0.01, *p < 0.05).

To explore the potential value of the risk scoring system in guiding individualized treatment, we systematically evaluated the sensitivity differences of patients in the high-risk and low-risk groups to eight commonly used anti-tumor drugs. For small molecule targeted therapeutic drugs, Dasatinib (a multi-target tyrosine kinase inhibitor), Saracatinib (Src family kinase inhibitor), HG-6-64-1 (Aurora kinase inhibitor), and WH-4-023 (Src/Lck dual inhibitor) showed significantly higher IC50 values in low-risk group patients (Figures 10A–D), indicating that the low-risk group has lower sensitivity to these drugs.

For traditional chemotherapy drugs, Bortezomib (proteasome inhibitor) and YM155 (survivin inhibitor) also showed significantly higher IC50 values in the low-risk group (Figures 10E,F). This suggested that high-risk tumors are more sensitive to proteasome pathway inhibition and apoptosis regulatory drugs. In contrast, Epothilone B (microtubule stabilizer) and Gemcitabine (nucleoside analog) showed lower IC50 values in the low-risk group (Figures 10G,H), indicating that the low-risk group has higher sensitivity to these two drugs. This may reflect the specific sensitivity of low-risk tumors to microtubule-targeting drugs and DNA synthesis inhibitors.

Comprehensive analysis showed that high-risk group patients are more sensitive to drugs such as Dasatinib, Saracatinib, HG-6-64-1, WH-4-023, Bortezomib, and YM155 that target tyrosine kinases, cell cycle regulation, proteasomes, and apoptosis pathways, while low-risk group patients show higher sensitivity to drugs such as Epothilone B and Gemcitabine that act on microtubule dynamics and nucleotide metabolism. This differential drug sensitivity pattern provides a molecular basis for individualized treatment plan selection for patients in different risk groups, reveals the intrinsic differences between high-risk and low-risk tumors in signaling pathway dependence and metabolic characteristics, and provides actionable guidance for treatment strategy formulation in the era of precision medicine.

### 3.10 Functional experimental validation of FCGR2A

**FIGURE 11:**
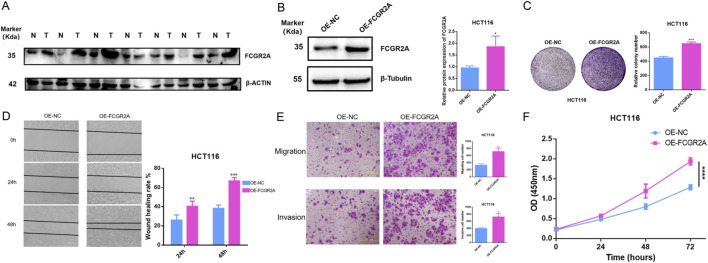
Experimental Validation of FCGR2A Function in Colon Cancer. **(A)** Western blot analysis of FCGR2A protein expression levels in colon cancer cell lines and normal colon epithelial cell lines, with β-ACTIN as an internal reference control. **(B)** Western blot validation of FCGR2A overexpression efficiency in HCT116 cells, with β-Tubulin as an internal reference. **(C)** Colony formation assay evaluating the effect of FCGR2A on HCT116 cell proliferation ability, with representative colony formation images on the left and quantitative statistical results on the right. **(D)** Wound healing assay detecting the effect of FCGR2A on cell migration ability, with representative images at 0, 24, and 48 h on the left and quantitative analysis of wound healing rate on the right. **(E)** Transwell assay evaluating the effect of FCGR2A on cell migration and invasion abilities, with representative staining images on the left and statistics of the number of cells that passed through the membrane on the right. **(F)** CCK-8 assay dynamically monitoring the effect of FCGR2A on HCT116 cell proliferation, with the blue line representing the OE-NC group and the pink line representing the OE-FCGR2A group. All data are expressed as mean ± SD, and statistical analysis was performed using Student’s t-test. (****p < 0.0001, ***p < 0.001, **p < 0.01, *p < 0.05).

Among the core genes of the constructed risk score model, FCGR2A has the largest weight coefficient, suggesting its most significant contribution to the model. Therefore, we first selected FCGR2A as a representative gene for in-depth functional validation, and subsequent studies will systematically validate other core genes. To verify the biological function of the key gene FCGR2A in colon cancer, we conducted a series of *in vitro* cell experiments. First, we detected the protein expression levels of FCGR2A in colon cancer cell lines and normal colon epithelial cell lines. Western blot results showed (Figure 11A) that the expression level of FCGR2A protein (molecular weight approximately 35 kDa) in colon cancer cell lines was significantly higher than in normal colon epithelial cell lines, with β-ACTIN (approximately 42 kDa) as an internal reference control showing uniform expression. This result confirmed the upregulated expression of FCGR2A in colon cancer cells at the protein level, consistent with the results of earlier bioinformatics analysis.

To further explore the function of FCGR2A, we constructed HCT116 cell lines with FCGR2A overexpression. Western blot validation showed (Figure 11B) that compared with the empty vector group, i.e., negative control group (OE-NC), FCGR2A protein expression was significantly upregulated in the FCGR2A overexpression group (OE-FCGR2A), with β-Tubulin (approximately 55 kDa) as an internal reference remaining stable. Quantitative analysis further confirmed that the relative expression level of FCGR2A protein in the OE-FCGR2A group was significantly higher than in the OE-NC group (*p* < 0.05), indicating successful construction of the overexpression model.

We evaluated the effect of FCGR2A on cell proliferation ability through colony formation assay (Figure 11C). The results showed that the number of colonies formed in the OE-FCGR2A group was significantly more than in the OE-NC group, with colony density and size also significantly increased. Quantitative analysis showed that the relative number of colonies formed in the OE-FCGR2A group was approximately 650, significantly higher than approximately 450 in the OE-NC group (*p* < 0.001). This result suggested that FCGR2A overexpression can significantly promote the proliferation and colony formation ability of colon cancer cells, playing an important role in the long-term survival and expansion of tumor cells.

Wound healing assay was used to evaluate the effect of FCGR2A on cell migration ability (Figure 11D). Observing wound healing at three time points of 0, 24, and 48 h, the cell migration rate in the OE-FCGR2A group was significantly faster than in the OE-NC group. Quantitative analysis showed that at 24 h, the wound healing rate of the OE-FCGR2A group was approximately 40%, significantly higher than approximately 25% in the OE-NC group (*p* < 0.01); at 48 h, the healing rate of the OE-FCGR2A group was approximately 65%, while the OE-NC group was only approximately 40% (*p* < 0.001). This indicated that FCGR2A overexpression can significantly enhance the migration ability of colon cancer cells.

Transwell migration and invasion assays further validated the pro-metastatic function of FCGR2A (Figure 11E). In the migration experiment, the number of cells that passed through the membrane pores in the OE-FCGR2A group was significantly more than in the OE-NC group (*p* < 0.01); in the invasion experiment, the number of cells that passed through the Matrigel matrix in the OE-FCGR2A group was also significantly higher than in the OE-NC group (*p* < 0.01). These results indicated that FCGR2A not only promotes active migration of cells but also enhances the degradation and invasion ability of cells on the extracellular matrix, which are key steps in tumor metastasis.

Finally, CCK-8 proliferation assay dynamically monitored the effect of FCGR2A on cell growth (Figure 11F). Measuring OD450 nm values at four time points of 0, 24, 48, and 72 h, the proliferation curve of the OE-FCGR2A group (pink line) was consistently higher than that of the OE-NC group (blue line). At 72 h, the OD450 nm value of the OE-FCGR2A group was approximately 2.0, significantly higher than approximately 1.3 in the OE-NC group (*p* < 0.0001), indicating that FCGR2A overexpression significantly promotes cell proliferation.

Integrating these experimental results, we confirmed that FCGR2A is highly expressed in colon cancer, and its overexpression can significantly promote the proliferation, migration, and invasion abilities of tumor cells, playing an important oncogenic role in the occurrence, development, and metastasis of colon cancer, providing experimental evidence for FCGR2A as a potential therapeutic target.

## 4 Discussion

By analyzing the differences in ICD scores among T-cell subsets at the single-cell level, we found that T cells with high ICD scores exhibited unique gene expression programs (Guo L. et al., 2024). These genes were significantly associated with tumor prognosis at the bulk transcriptome level, suggesting that cell-level immunogenic features can be extrapolated to tissue-level risk stratification (Zhang et al., 2025). WGCNA further confirmed that these genes do not act in isolation but regulate immune responses through co-expression networks. The strong correlation between the blue module genes and the immune score (*r* = 0.92, *p* < 0.001) revealed the existence of systemic immune reprogramming (Yang et al., 2021). Methodologically, the systematic comparison of 117 algorithmic combinations surpassed the limitations of previous studies that relied on single or few algorithms (Qiu et al., 2020). RSF ultimately prevailed not only because of its ensemble learning nature but also due to its ability to capture high-order gene-gene interactions, which is crucial for understanding the synergistic mechanisms of ICD-related genes (Wang and Li, 2017; Bohannan et al., 2022). The immune microenvironment differences revealed by risk stratification have profound therapeutic implications (Duan and Luo, 2021). The immunosuppressive phenotype (M2 macrophage and Treg enrichment) in the high-risk group explains why such patients may derive limited benefit from immune checkpoint inhibitors (Mantovani and Locati, 2013; Brom et al., 2022). Conversely, the increased sensitivity to targeted drugs such as Dasatinib provides a theoretical basis for combination therapy strategies in specific molecular contexts (Perez et al., 2016). As a member of the Fc receptor family, FCGR2A overexpression leads to multifunctional enhancement, which not only validates the oncogenic role of this model gene but also suggests that it may contribute to immune evasion by modulating antibody-dependent cell-mediated cytotoxicity (ADCC) (Zhang et al., 2007). This finding offers a new therapeutic target for developing specific intervention strategies. The nomogram model organically integrates molecular features with traditional clinical parameters. Its C-index improvement to 0.823 indicates that biomarkers can significantly enhance the predictive power of existing staging systems (Jiang et al., 2024). This integrative strategy aligns more closely with the multifactorial decision-making approach in clinical practice.

Several studies have developed prognostic models based on autophagy-related genes (C-index: 0.796), ferroptosis-related genes (C-index: 0.838), and other immune-related signatures (Zhou et al., 2020; Wang et al., 2021; Liu et al., 2024). In comparison, our ICD-based signature demonstrated superior performance with a C-index of 0.968 in the TCGA training cohort and maintained robust predictive accuracy in independent validation cohorts (C-index: 0.767 and 0.855). This improvement can be attributed to the systematic integration of single-cell and bulk transcriptomics, comprehensive machine learning algorithm optimization, and the biological rationale that ICD captures the critical nexus linking tumor cell death, immune activation, and therapeutic response, providing a more holistic representation of tumor biology than single-pathway-focused models.

The FCGR2A gene encodes the low-affinity immunoglobulin G (IgG) Fc receptor IIa (CD32a), a transmembrane glycoprotein widely expressed on myeloid cells. It mediates ADCC and antibody-dependent cellular phagocytosis (ADCP) by recognizing the Fc fragment of IgG antibodies (de Taeye et al., 2020). In this study, FCGR2A was identified as the top among 15 core prognostic genes, being significantly upregulated in the high-risk group. *In vitro* functional experiments confirmed that FCGR2A overexpression significantly enhanced the proliferation, migration, and invasion abilities of HCT116 cells, directly validating its tumor-promoting role (Lu et al., 2024). Immune infiltration analysis showed that FCGR2A expression was significantly positively correlated with M2 macrophage infiltration (*p* < 0.001), suggesting that it may promote tumor progression by reshaping an immunosuppressive microenvironment. Previous studies mainly focused on the predictive value of FCGR2A as a biomarker for antibody therapy efficacy in breast and ovarian cancers, such as the FCGR2A-H131R polymorphism influencing trastuzumab treatment response, while functional studies in colon cancer remain scarce (Verschoor et al., 2024; Gavin et al., 2017).

While our functional experiments confirmed the oncogenic role of FCGR2A in colon cancer cells, the underlying molecular mechanisms warrant further investigation. Although FCGR2A is traditionally recognized as an Fc receptor on immune cells, emerging evidence suggests its expression on tumor cells may activate intrinsic oncogenic signaling independent of immune interactions (Guilliams et al., 2014). Previous studies have demonstrated that Fc receptors can trigger PI3K/AKT and MAPK/ERK pathways, which are central regulators of cell survival, proliferation, and migration in cancer (Shapiro et al., 2017). We hypothesize that FCGR2A overexpression may constitutively activate these pathways through ligand-independent receptor oligomerization and immunoreceptor tyrosine-based activation motif (ITAM) phosphorylation, or through cross-talk with receptor tyrosine kinases such as EGFR. Future mechanistic studies employing phosphoproteomics and pathway inhibition experiments will be essential to dissect the precise signaling networks activated by FCGR2A in colon cancer.

Deep analysis of the tumor immune microenvironment revealed fundamental differences in immune regulatory networks between high- and low-risk groups. The high-risk group was enriched with M2 macrophages and Tregs, while the low-risk group was characterized by activated CD4^+^ T cells and plasma cell infiltration (Fu et al., 2024; Li et al., 2021). However, these immune phenotypic differences are not static changes in cellular composition but reflect dysregulation of dynamic immune regulatory circuits. Traditionally, M2 macrophages have been regarded merely as immunosuppressive cells (Hong et al., 2024). Yet, the strong correlation between FCGR2A and M2 macrophages (*r* = 0.61) revealed a more complex mechanism. FCGR2A may not only passively mark M2 macrophage infiltration but also actively participate in the positive feedback regulation of macrophage polarization. Tumor cells with high FCGR2A expression recruit and polarize M2 macrophages, which in turn secrete IL-10 and TGF-β to upregulate FCGR2A expression in tumor cells, forming a self-reinforcing immunosuppressive loop. This finding aligns with Mantovani et al. (2022) research on the plasticity of tumor-associated macrophages, but we further demonstrated the driving role of ICD-related genes in this process. More importantly, GSEA revealed concurrent activation of epithelial-mesenchymal transition (EMT) and angiogenesis pathways in the high-risk group. This co-activation pattern suggests that immune suppression, tissue remodeling, and metabolic reprogramming constitute an interdependent triangular relationship (Xia et al., 2021). De Palma et al. reported that abnormal tumor vasculature not only physically blocks immune cell infiltration but also that endothelial cells expressing immune checkpoint molecules can actively suppress T-cell function (De Palma et al., 2013). Our findings indicate that activation of oxidative phosphorylation pathways in the low-risk group may help maintain immune cell activity by reducing the accumulation of immunosuppressive metabolic byproducts such as lactate. This metabolism-immune coupling mechanism has been rarely reported in previous colon cancer studies. A deeper distinction lies in the evolutionary stages of immune editing. The plasma cell enrichment in the low-risk group suggests involvement of humoral immunity, echoing the findings of Shalapour et al. on the anti-tumor effects of IgA + plasma cells (Shalapour et al., 2017). However, the high-risk group may have already entered the immune escape phase. Even with immune cell infiltration (e.g., T cells recruited by CCL5 overexpression), they may become functionally inactivated due to checkpoint molecule expression and metabolic suppression. The transition from immune surveillance to immune escape is not a linear process but the result of multiple positive and negative feedback loops regulated by ICD genes. This provides a systems biology perspective for understanding immune therapy resistance.

Notably, the identified drugs have clinical translation potential. Dasatinib, showing higher sensitivity in the high-risk group, is currently being evaluated in clinical trials for advanced colorectal cancer and other solid malignancies (NCT00388427, NCT01652976). Gemcitabine, to which the low-risk group showed higher sensitivity, has been investigated for metastatic colorectal cancer (NCT00007943, NCT00220155). Bortezomib has been explored in early-phase colorectal cancer trials. These findings suggest that our risk stratification system could guide patient selection for these agents in biomarker-driven clinical trials. The increased sensitivity of the high-risk group to SRC kinase inhibitors is related to EMT pathway activation, suggesting the establishment of a translational pipeline from risk score guidance to patient-derived organoid validation and then to clinical medication (Yang et al., 2022). By performing personalized drug sensitivity screening in organoid co-culture systems that retain immune microenvironment components, precise linkage from biomarker identification to therapeutic decision-making can be achieved (Ning et al., 2025). More importantly, differences in drug sensitivity can optimize existing therapeutic combinations. The low-risk group’s sensitivity to nucleoside analogs stems from its oxidative phosphorylation metabolic pattern, suggesting that such immune-activated patients are suitable for combining chemotherapy-induced ICD with immune checkpoint inhibitors. Conversely, the high-risk group’s angiogenesis pathway activation and immunosuppressive microenvironment characteristics suggest a sequential strategy: first using targeted drugs to remodel the microenvironment, followed by the introduction of immunotherapy. Such temporally ordered interventions may be more effective than simple combination therapy in overcoming immunosuppressive barriers.

The clinical value of the nomogram model goes beyond static prognosis prediction. Its ability to integrate molecular characteristics with anatomical staging provides a new dimension for therapeutic decision-making. The prognostic value of the risk score independent of TNM staging implies that patients with the same stage may have distinct outcomes due to differences in immune microenvironmental states. This directly guides individualized adjustments in adjuvant therapy intensity—stage II high-risk patients should consider aggressive interventions, while stage III low-risk patients may appropriately de-escalate treatment intensity. A more forward-looking application lies in dynamic risk management. By regularly monitoring key ICD gene expression or molecular feature changes in circulating tumor DNA postoperatively, a comprehensive management process can be established—from baseline risk assessment to continuous monitoring and adaptive therapeutic adjustment (Guo Y. et al., 2024).

This study still has several limitations that need improvement in future work. First, all analyses were based on retrospective data from public databases, necessitating validation of the prognostic model’s practical utility through prospective multicenter clinical trials. Regarding model performance, our model demonstrated a high C-index of 0.968 in the training set but decreased to 0.767–0.855 in external validation, reflecting common cross-platform biomarker validation challenges. This gap stems from platform differences (RNA-sequencing *versus* microarray), patient heterogeneity, and batch effects rather than overfitting, as evidenced by our rigorous 10-fold cross-validation and completely independent external validation sets. Importantly, our model outperformed existing colorectal cancer prognostic signatures including CMS, ColoGuideEx, and Oncotype DX when compared in the same validation cohorts, supporting its clinical value. Future validation in prospective multicenter cohorts and platform-specific optimization will further enhance generalization performance. Second, although FCGR2A underwent functional validation, the specific mechanisms and regulatory relationships of the other 14 key genes remain to be elucidated. Third, this study focused on the transcriptomic level, while protein modifications, metabolomic profiles, and epigenetic landscapes also influence the ICD process. Fourth, validation in only one cell line cannot establish generalizability across the molecular heterogeneity of colon cancer (MSI status, driver mutations, CMS subtypes). The absence of loss-of-function experiments (knockout/knockdown) limits our ability to demonstrate FCGR2A’s necessity for malignant phenotypes, and the lack of *in vivo* studies prevents assessment of its role in the tumor microenvironment. We are currently conducting comprehensive follow-up studies including multi-cell line validation, bidirectional functional experiments, and *in vivo* tumorigenesis and metastasis models in immunocompetent mice to fully elucidate FCGR2A’s therapeutic potential. Fifth, tumor evolutionary dynamics are key factors affecting prognosis and therapeutic response. Longitudinal sampling at multiple time points before and after treatment combined with single-cell multi-omics sequencing can track the evolutionary trajectory of ICD gene features under therapeutic pressure, thereby developing dynamic risk assessment systems to adjust treatments in real time. Sixth, the data in this study were predominantly from European and American populations, while colon cancer exhibits significant molecular heterogeneity and prognostic differences across races. Independent validation in Asian, African, and other multi-ethnic cohorts is urgently needed to verify the model’s generalizability. Finally, a deeper limitation lies in the uncertainty of causal inference. Although associations between ICD genes and prognosis were identified, correlation does not imply causation. *In vitro* cell line experiments cannot fully reproduce the complexity of *in vivo* tumor–host interactions. Whether high expression of these genes drives tumor progression or results from tumor microenvironment remodeling remains to be distinguished through genetic perturbation experiments and temporal causal analysis methods. Further validation in immunocompetent syngeneic or humanized mouse models is required. In the future, the introduction of artificial intelligence and deep learning algorithms may overcome the limitations of traditional statistical approaches. By integrating pathological images, radiological features, and molecular data through convolutional neural networks, multi-modal prognostic prediction systems can be constructed. The most translationally promising direction lies in developing key ICD genes as therapeutic targets. Designing small-molecule inhibitors or monoclonal antibodies against core molecules such as FCGR2A and evaluating their anti-tumor efficacy and immune microenvironment remodeling potential in organoid and patient-derived xenograft models could open new avenues for targeted immunotherapy in colon cancer.

## 5 Conclusion

We integrated single-cell and bulk transcriptomic data with clinical information to characterize ICD heterogeneity in colon cancer and develop an ICD-related prognostic model. Using ssGSEA-based ICD scoring, WGCNA screening, and systematic machine-learning evaluation, we constructed a 15-gene ICD signature with strong prognostic performance in TCGA and stable validation in independent GEO cohorts. Risk stratification captured distinct immune microenvironment features, pathway activities (including EMT and angiogenesis in high-risk tumors), and predicted drug sensitivities, supporting its value for individualized treatment planning. Functional assays further showed that FCGR2A promotes colon cancer cell proliferation, migration, and invasion *in vitro*. Overall, this ICD-based framework may aid prognosis prediction and therapeutic stratification, but requires prospective and mechanistic validation.

## Data Availability

The original contributions presented in the study are publicly available. This data can be found here: TCGA-COAD (https://portal.gdc.cancer.gov/), GEO (GSE17538, GSE38832) (https://www.ncbi.nlm.nih.gov/geo/), GTEx (https://gtexportal.org/), ImmPort (https://www.immport.org/),and InnateDB (https://www.innatedb.com/). Further inquiries can be directed to the corresponding author(s).
