# Supplementary material for: Multi-omics integration and machine learning-driven construction of an immunogenic cell death prognostic model for colon cancer and functional validation of FCGR2A
Source: Front Pharmacol. 2026 Jan 22;16:1746907. doi: 10.3389/fphar.2025.1746907 (PMC12872855; doi:10.3389/fphar.2025.1746907)
Supplement: Supplementary file 1 [file DataSheet1.docx]

ICD-genes

FOXP3

IFNB1

CXCR3

IFNG

P2RX7

IL6

CASP8

CD8B

PIK3CA

PRF1

NT5E

EIF2AK3

CD8A

ENTPD1

IL17RA

TLR4

MYD88

ATG5

IL1R1

CASP1

IFNGR1

BAX

CD4

NLRP3

LY96

IL10

TNF

PDIA3

CALR

HMGB1

IL1B

HSP90AA1
